# Protecting Health from Climate Change: Preparedness of Medical Interns

**DOI:** 10.4103/0970-0218.58390

**Published:** 2009-10

**Authors:** Jai Pal Majra, Das Acharya

**Affiliations:** Department of Community Medicine, K.S. Hegde Medical Academy, Mangalore, India

**Keywords:** Climate change, health protection, preparedness, medical interns

## Abstract

**Context:**

Climate change is a significant and emerging threat to public health and to meet the challenge, health systems require qualified staff.

**Aims:**

To study the preparedness of medical interns to meet the challenge of protecting health from climate change.

**Settings and Design:**

Medical colleges in a coastal town. Cross-sectional study.

**Materials and Methods:**

A proportionate number of medical interns from five medical colleges were included in the study. Level of awareness was used as a criterion to judge the preparedness. A self-administered, pretested, open-ended questionnaire was used. Responses were evaluated and graded.

**Statistical Analysis Used:**

Proportions, percentage, Chi-test.

**Results:**

About 90% of the medical interns were aware of the climate change and human activities that were playing a major role. Ninety-four percent were aware of the direct health impacts due to higher temperature and depletion in ozone concentration, and about 78% of the respondents were aware about the change in frequency / distribution of vector-borne diseases, water borne / related diseases, malnutrition, and health impact of population displacement. Knowledge regarding health protection was limited to mitigation of climate change and training / education. Options like adaptation, establishing / strengthening climate and disease surveillance systems, and health action in emergency were known to only nine (7%), eight (6%), and 17 (13%), respectively. Collegewise difference was statistically insignificant. Extra / co-curricular activities were the major source of knowledge.

**Conclusions:**

Majority of medical interns were aware of the causes and health impacts of climate change, but their knowledge regarding health protection measures was limited.

## Introduction

Our personal health may seem to relate mostly to prudent behavior, heredity, occupation, local environmental exposures, and health-care access, but health of the sustained population requires the life supporting “services” of the biosphere. Populations of all animal species depend on supplies of food and water, freedom from excess infectious disease, and the physical safety and comfort conferred by climatic stability. The world's climate system is fundamental to this life support. Humankind's activities are altering the world's climate by increasing the atmospheric concentration of energy-trapping gases (greenhouse gases), thereby amplifying the natural “greenhouse effect” that makes the Earth habitable. These greenhouse gases (GHGs) comprise principally of carbon dioxide (mostly from fossil fuel combustion and forest burning), plus other heat-trapping gases such as methane (from irrigated agriculture, animal husbandry, and oil extraction), nitrous oxide, and various human-made halocarbons. Over the last 50 years the world's average surface temperature has increased by approximately 0.65°C. There is strong evidence that most of the warming observed is attributable to human activities.(1) Climatologists forecast further warming, along with changes in precipitation and climatic variability, during the coming century and beyond. This is likely to have an impact on human health. Some of these health impacts would be beneficial. For example, milder winters would reduce the seasonal winter-time peak in deaths that occur in temperate countries, while in the currently hot regions a further increase in temperatures might reduce the viability of disease-transmitting mosquito populations. Overall, however, scientists consider that most of the health impacts of climate change would be adverse.(2) Climatic changes over recent decades have probably already affected some health outcomes. The climate change was estimated to be responsible in 2000, for approximately 2.4% of worldwide diarrhoea, and 6% of malaria in some middle-income countries and was estimated to have caused 150,000 deaths and 5.5 million DALYS in the year 2000.(3)

The impacts of climate on human health will not be evenly distributed around the world, the vulnerability of a population will also depend on factors such as, pre-existing health status, and the quality and availability of public healthcare apart from local environmental conditions and other socio-economic factors.(4) This topic is emerging as a major theme in population health research, social policy development, and advocacy. As health systems are labor-intensive and require qualified and experienced staff, the present study was conducted to discern the preparedness of the medical interns (future doctors) to meet the challenge of protecting health from climate change.

## Materials and Methods

The present study was carried in a coastal town in Karnataka state in India. The town hosts five medical colleges under two different universities with a total annual intake capacity of 650 students for MBBS. Medical interns from all the medical colleges in the town were included in the study. Cluster sampling technique was used and each college was taken as a cluster. The interns were posted in different departments, including outreach postings, with different time schedules. Therefore, from each cluster, a proportionate number of subjects (20%) who first came in contact with the investigators and gave their consent were included in the study. Each participant was given a self-administered, pre-tested, open-ended questionnaire to solve on the spot. The purpose of the study and all the terms used in the study were explained to the respondents and they were ensured that total confidentially would be maintained. Level of awareness was used as a criterion to judge the preparedness of the medical interns to protect the health from climate change. The questionnaire was divided into two sections. Section one contained questions related to climatic change and the human activities that led to climatic change, and section two contained questions on the effects of climatic change on human health and ways to protect human health from climatic change. Responses thus received were evaluated and graded using the five-point scale. Respondents securing < 20%,20 – 40%, 40 – 60%, 60 – 80%, and more than 80% points were graded as having poor, fair, good, very good, and excellent level of preparedness, respectively. Collegewise differentials were also analyzed. Proportions, percentage, and Chi-test were used to analyze the data.

## Results

This study represents 650 medical interns from five medical colleges, affiliated to two different universities. Sample size was 130. Response rate was cent percent for colleges as well as respondents. All the medical interns were aware that climate change was occurring and human activities were playing a major role in this climate change, by increasing the atmospheric concentration of energy-trapping gases, thereby, amplifying the natural “greenhouse effect” that made the Earth habitable. Boys were more aware than girls, although the difference was not statistically significant (*P* value > 0.05). All the respondents were aware that climate change was leading to climatic extremes, simple (very high and very low temperatures) as well as complex events like floods, droughts, hurricanes, and so on. A majority 113 (87%), 124 (95%), and 120 (92%) of the respondents were aware that climate change would also be manifested as a rise in mean sea level leading to coastal floods forcing displacement of the coastal population, more variable precipitation in the form of change in distribution and intensity of rainfall, and degrading of ecosystems and loss of biodiversity, respectively. A majority of the respondents were aware of the various human activities, such as, the ever increasing population (85%), industrialization (88%), urbanization (92%), deforestation (92%), increase in international trade/travel (85%), and our increasing dependence on carbon-based energy, such as fossil fuels (88%), were contributing to the climate change. Most of the respondents, 122 (94%) were aware of the direct health impacts [Table 1], such as, increased morbidity and mortality due to higher temperature and increase in prevalence of sunburns, skin cancers 92%) and so on, due to depletion in the ozone concentration. However, about three fourth of the respondents were aware of the more important health impacts, such as, the change in frequency/distribution of vector-borne diseases, water borne/related diseases, malnutrition, and health impact of population displacement, due to floods (coastal and inland) and droughts, and so on. None of the respondents mentioned the decrease that would occur in morbidity and mortality due to milder winters and a further increase in temperatures, in the currently hot regions, might reduce the viability of disease-transmitting mosquito populations, and hence, diseases transmitted by mosquitoes.

**Table 1:** Health impacts of climate change

Health impacts	Correct responses n = 130
Direct physical impact of extreme climatic events	122 (94)
Change in frequency / distribution of vector-borne diseases	101 (78)
Water borne / related diseases	98 (75)
Health impact of population displacement	101 (78)
Malnutrition	101 (78)
Impact of ozone depletion on skin, eye, immune system, etc.	120 (92)

Figures in parenthesis are in percentages

Knowledge of the medical interns regarding health protection [Table 2] was limited to mitigation of climate change by means of decreasing the use and dependency on fossil fuels, more use of renewable sources of energy, decreasing air pollution, reforestation, and so on, training of the healthcare provider, and education of the masses.

**Table 2:** Health protection measures

Health protection measures	Correct responses n = 130
Mitigation of climatic change	120 (92)
Adaptation	9 (7)
Strengthening climate and disease	8 (6)
surveillance systems	
Health action in emergencies	17 (13)
Education and communication	124 (95)

Figures in parenthesis are in percentages

Options like adaptation, that is, adjustment in response to actual or expected climatic stimuli or their effects, which moderates harm or exploits beneficial opportunities, establishing/strengthening climate and disease surveillance systems, and health action in emergency, were known to only nine (7%), eight (6%), and 17 (13%) respondents, respectively. Collegewise difference in the level of awareness was also observed [Table 3] but this difference was not statistically significant (*P* value > 0.05). Extra/co-curricular activities were the major source of knowledge for the medical interns.

**Table 3:** Institutional differentials in level of preparedness of medical interns

College	Level of preparedness					Total n = 130
		
	Poor	Fair	Good	Very good	Excellent	
A	2 (10)	1 (5)	1 (5)	15 (75)	1 (5)	20 (100)
B	2 (10)	4 (20)	2 (10)	12 (60)	0 (0)	20 (100)
C	1 (5)	3 (15)	4 (20)	12 (60)	0 (0)	20 (100)
D	4 (20)	10 (50)	5 (25)	1 (5)	0 (0)	20 (100)
E	2 (4)	5 (10)	3 (6)	35 (70)	5 (10)	50 (100)
Total	11 (8)	23 (18)	15 (11)	75 (58)	6 (5)	130 (100)

Figures in parenthesis are in percentages

## Discussion

Climate change is a significant and emerging threat to public health; hence it is finding an increasingly central position on the international agenda, as most recently evidenced by the Nobel Prize awarded to former US Vice President Al Gore and a team of UN experts, for their work on the subject. There is strong evidence that most of the warming observed over the last 50 years is attributable to human activities.(5) During the twentieth century, the world's average surface temperature has increased by approximately 0.6°C and approximately two-thirds of that warming has occurred, since 1975. As we continue to change atmospheric composition, global average surface temperature will rise by 1.4 to 5.8°C in this century, along with changes in precipitation and other climatic variables.(4) Extremes of temperature can kill. In many temperate countries, death rates during the winter season are 10 – 25% higher than those in the summer. In July 1995, a heat-wave in Chicago, US, caused 514 heat-related deaths (12 per 100,000 population) and 3300 excess emergency admissions. Most of the excess deaths during times of thermal extreme are in persons with pre-existing disease, especially cardiovascular and respiratory disease. The very old, the very young, and the frail are most susceptible.(6) Climatic factors are an important determinant of various vector-borne diseases, many enteric illnesses and certain water-related diseases. The El Niño phenomenon provides an analog for understanding the future impacts of global climate change on infectious diseases.(6) The disease's sensitivity to climate is illustrated by desert and highland fringe areas where higher temperatures and / or rainfall, associated with El Niño, may increase transmission of malaria.(7) Between 1970 and 1995, the annual number of dengue epidemics in the South Pacific was positively correlated with La Niña conditions (i.e., warmer and wetter).(8) Certain rodent-borne diseases are associated with flooding, including leptospirosis, tularaemia, and viral hemorrhagic diseases. Other diseases associated with rodents and ticks, and which show association with climatic variability include, Lyme disease, tick-borne encephalitis, and Hantavirus pulmonary syndrome.(9) Many diarrhoeal diseases vary seasonally, suggesting sensitivity to climate. Both floods and droughts increase the risk of diarrhoeal diseases. Major causes of diarrhoea linked to heavy rainfall and contaminated water supplies are: cholera, cryptosporidium, *E.coli* infection, giardia, shigella, typhoid, and viruses such as hepatitis A.(9) Globally, the impact of weather disasters (droughts, floods, storms, and bushfires) have been increasing. El Niño events influence the annual toll of persons affected by natural disasters.(10) Globally, disasters triggered by droughts occur especially during the year after the onset of El Niño, and climate change is likely to increase the frequency and / or amplitude of El Niño.(5) Stratospheric ozone depletion is expected to cause an increase in skin cancer incidence in fair-skinned populations living in mid to high latitudes(11,12) and may also influence the occurrence and progression of various autoimmune diseases.(13) It illustrates well, how climatic extremes can affect human health. Climate change already contributes to the global burden of disease, and this contribution is expected to grow in the future.(3) In 2030, the estimated risk of diarrhoea will be up to 10% higher in some regions than if no climate change occurred. The European population will experience an approximate 5% and the US population a 10% excess of total skin cancer incidence, by 2050. Health professionals are on the front line in dealing with the impacts of climate change. Fortunately, much of the health risk is avoidable through existing health programs and interventions. Response options include the mitigation of greenhouse gases that provides a mechanism for slowing, and perhaps eventually halting, the buildup of greenhouse gases in the atmosphere. Adaptation is the other option. The term “adaptation,” used by the climate change community is analogous to the concept of prevention used in public health.(14) The rebuilding and maintaining of public health infrastructure is often viewed as the “most important, cost-effective, and urgently needed” adaptation strategy.(15) This includes public health training, more effective surveillance and emergency response systems, and sustainable prevention and control programs. The present study reveals that this is the least known area to the medical interns as far as protecting health from climate change is concerned. These findings are largely consistent with a recently released study of the local Public Health Department directors in California,(16) and a nationally representative survey of local Health Department directors in the US.(17) Most respondents felt that personnel in their health department had a lack of knowledge about the health effects of climate change and expertise to create an effective mitigation or adaptation plan. This may be because of the fact that the main source of information on the subject, as cited by the respondents in our study, is extra / co-curricular activities, which may be concentrating on the mitigation only. Collegewise differences point toward unequal exposure that may be due to the differences in the extra / co-curricular activities organized in the respective colleges.

## Conclusion and Recommendations

Climate change is a significant and emerging threat to public health. A majority of medical interns are aware about the causes and health impacts of climate change, but their knowledge regarding health protection measures is limited. This may be due to the insufficient space assigned to the subject in the medical curriculum. Therefore, it is recommended that learning resource materials on the subject are developed and inducted into the medical curriculum.

### Limitations of the study

The sampled respondents were selected on a first-come basis and on their willingness to participate in the study. This may have induced some bias in the study sample.
